# Predictive values of multiple non-invasive markers for myocardial fibrosis in hypertrophic cardiomyopathy patients with preserved ejection fraction

**DOI:** 10.1038/s41598-021-83678-z

**Published:** 2021-02-22

**Authors:** Yumin Li, Jia Liu, Yukun Cao, Xiaoyu Han, Guozhu Shao, Xiaoyue Zhou, Jin Gu, Tong Liu, Yue Cui, Heshui Shi

**Affiliations:** 1 Department of Radiology, Union Hospital, Tongji Medical College, Huazhong University of Science and Technology, 1277 JieFang Avenue, Wuhan, 430022 China; 2 Hubei Province Key Laboratory of Molecular Imaging, Wuhan, 430022 China; 3 MR Collaboration, Siemens Healthineers Ltd, Shanghai, 201318 China

**Keywords:** Cardiology, Biomarkers, Diagnostic markers

## Abstract

Myocardial fibrosis assessed by late gadolinium enhancement (LGE) on cardiovascular magnetic resonance (CMR) is associated with cardiovascular outcomes in hypertrophic cardiomyopathy (HCM) patients, but little is known about the utility of non-invasive markers for detecting LGE. This study aims to explore the association between cardiac-specific biomarkers, CMR myocardial strain, left ventricular (LV) hypertrophy and LGE in HCM patients with preserved ejection fraction (EF) and investigate the predictive values of these indexes for LGE. We recruited 33 healthy volunteers and 86 HCM patients with preserved EF to undergo contrast-enhanced CMR examinations. In total, 48 of 86 HCM patients had the presence of LGE. The LGE-positive patients had significant higher serum high-sensitivity cardiac troponin I (hs-cTnI) and N-terminal pro b-type natriuretic peptide (Nt-proBNP) levels and lower global longitudinal (GLS) and circumferential (GCS) strains than the LGE-negative group. The LGE% was independently associated with the Nt-proBNP levels, GCS, LV end-diastolic maximum wall thickness (MWT) and beta-blocker treatment. In the receiver operating characteristic curve analysis, the combined parameters of Nt-proBNP ≥ 108.00 pg/mL and MWT ≥ 17.30 mm had good diagnostic performance for LGE, with a specificity of 81.25% and sensitivity of 70.00%. These data indicate that serum Nt-proBNP is a potential biomarker associated with LGE% and, combined with MWT, were useful for identifying myocardial fibrosis in HCM patients with preserved EF. Additionally, LV GCS may be a more sensitive indicator for reflecting the presence of myocardial fibrosis than GLS.

## Introduction

Hypertrophic cardiomyopathy (HCM) is the most common inherited cardiomyopathy and its pathological features manifest as cardiac myocyte hypertrophy, disarray, and fibrosis^1,2^. Myocardial fibrosis in HCM patients has become an important clinical problem and might lead to arrhythmias, sudden cardiac death (SCD) and even advanced clinical events^3,4^. Extensive late gadolinium enhancement (LGE) imaging on cardiovascular magnetic resonance (CMR) is currently recognized as the gold standard for the identification of left ventricular (LV) focal replacement fibrosis^5^. Data currently suggest that LGE is highly promising in predicting SCD and progression to heart failure in HCM^6,7^. However, the administration of contrast agents needed for this technology may result in systemic nephrogenic sclerosis^8^, and CMR examination is expensive and has many contraindications. Notably, evidences from experimental studies in genetically modified animal models of HCM showed that treatment with pharmacological agents have the potential to reverse or attenuate established cardiac fibrosis^9,10^. Therefore, identifying the non-invasive biomarkers for the early detection and prediction of myocardial fibrosis could have a role in management and risk stratification in HCM.

Currently, cardiac-specific biomarkers, particularly N-terminal pro b-type natriuretic peptide (Nt-proBNP) and high-sensitivity cardiac troponin I (hs-cTnI), have played a key role in the diagnosis, treatment and risk stratification in the cardiovascular field^11–13^. The two biomarkers are widely used in daily clinical practice due to their easy acquisition, cost-effectiveness, high reproducibility and no contraindications. Although some modest sized studies have been showed a correlation of fibrosis with hs-cTnI and Nt-proBNP^14,15^, the prognostic value of both biomarkers in predicting fibrosis in HCM patients was inconsistent and, not systematically evaluated in HCM patients with preserved ejection fraction (EF). Additionally, CMR tissue-tracking technology can evaluate myocardial contractile abnormalities with rapid post-processing for routine cine images^16^, which was widely used in various cardiovascular diseases^17–19^. There are some studies showing close correlation between myocardial mechanics and fibrosis in HCM, as well as associations with ventricular arrhythmias^20,21^.

Our study, therefore, used advanced CMR tissue tracking technology and LGE analysis to explore the association between ﻿cardiac-specific biomarkers, myocardial strain, wall thickness and LGE (%) in HCM patients with preserved EF and investigate the diagnosis performance of these indexes for myocardial fibrosis.

## Results

### Baseline clinical and biochemical characteristics

**Figure 1:**
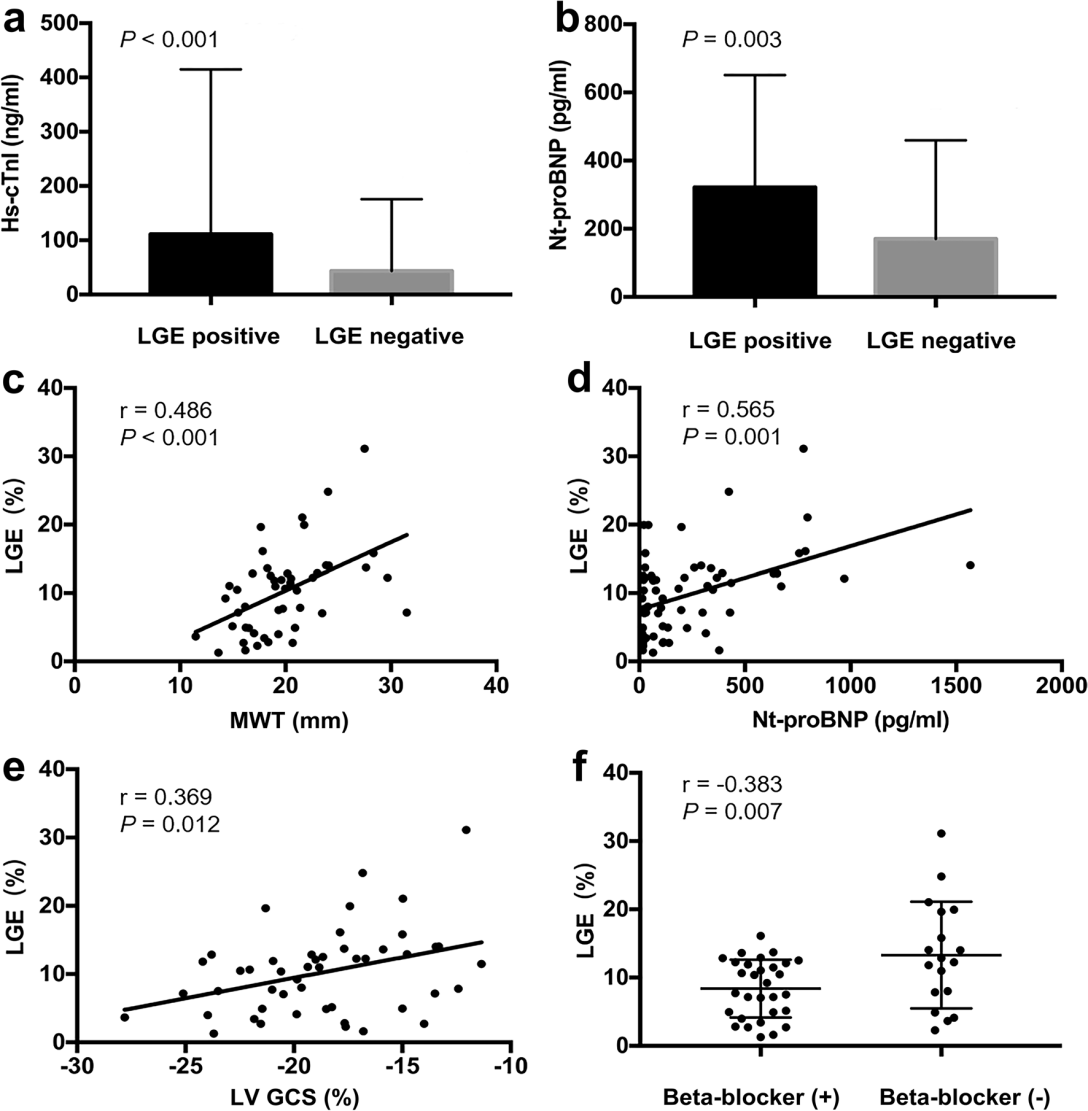
Comparison of the mean serum hs-cTnI (**a**) and Nt-proBNP (**b**) levels between LGE-positive and LGE-negative patients. Correlations between the LGE% and the MWT (**c**), Nt-proBNP (**d**), GCS (**e**) and beta-blocker treatment (**f**) in HCM patients. hs-cTnI high-sensitivity cardiac troponin I, Nt-proBNP N-terminal pro B-type natriuretic peptide, LGE late gadolinium enhancement, MWT maximum wall thickness, GCS global circumferential strain.

The baseline characteristics of the 86 HCM patients (48 subjects with LGE, 38 subjects without LGE) and 33 controls are summarized in Table 1. The LGE-negative patients had a significantly higher heart rate than the controls and LGE positive patients (*p* < 0.01). The LGE-positive patients had a trend towards a higher prevalence of diabetes mellitus (*p* = 0.058), a higher use of diuretics (*p* = 0.051) and trimetazidine (*p* = 0.083) than the LGE-negative group. The serum hs-cTnI, Nt-proBNP and creatine kinase-MB (CK-MB) levels and the proportions of patients with Nt-proBNP values of  > 100.00 pg/ml were significantly higher in LGE-positive patients than in LGE-negative patients (*p* < 0.05 for all) (Fig. 1, a and b). There were no significant differences in any other characteristics of the study population.

**Table 1:** Demographic and clinical characteristics of the study population.

Variables	Controls (n = 33)	All HCM (n = 86)	*p* value	LGE negative (n = 38)	LGE positive (n = 48)	*p’* value
**Demographics**						
Age (years)	47.85 ± 10.37	52.81 ± 11.92	0.316	51.71 ± 13.16	53.69 ± 10.90	0.085
Male (n, %)	25 (75.76)	70 (81.40)	0.610	32 (84.22)	38 (79.17)	0.668
Height (cm)	168.27 ± 6.00	169.67 ± 6.22	0.637	169.66 ± 6.84	169.71 ± 5.76	0.538
Weight (kg)	68.46 ± 8.76	71.79 ± 10.53	0.436	71.76 ± 10.44	71.81 ± 10.71	0.278
Body mass index (kg/m^2^)	24.17 ± 2.80	24.86 ± 2.94	0.746	24.83 ± 2.58	24.88 ± 3.22	0.517
Body surface area (m^2^)	1.79 ± 0.13	1.85 ± 0.16	0.294	1.85 ± 0.16	1.84 ± 0.17	0.197
**Clinical characteristics, n, %**						
Heart rate (bpm)	64.39 ± 10.23	69.81 ± 10.83	0.066	71.32 ± 9.71*	68.63 ± 11.61^#^	0.009
LVOTG at rest (mmHg)	–	6.70 (4.00 − 25.04)	–	5.30 (3.24 – 22.36)	10.24 (4.00–27.04)	0.122
Obstructive	0 (0)	34 (39.53)	–	12 (31.58)	22 (45.83)	0.192
Nonobstructive	33 (33)	52 (60.47)	–	26 (68.42)	26 (54.17)	0.192
NYHA functional class I	0 (0)	48 (55.81)	–	26 (68.42)	22 (45.83)	–
NYHA functional class II	0 (0)	27 (31.40)	–	8 (21.05)	19 (39.58)	–
NYHA functional class III	0 (0)	11 (12.79)	–	4 (10.53)	7 (14.58)	–
Hypertension	0 (0)	36 (41.86)	–	16 (42.11)	20 (41.67)	1.000
Diabetes	0 (0)	12 (13.95)	–	2 (5.56)	10 (20.83)	0.058
﻿Hyperlipidemia	0 (0)	20 (23.26)	-	9 (23.68)	11 (22.92)	1.000
﻿Smoker	10 (30.30)	41 (47.67)	0.086	16 (42.11)	25 (52.08)	0.391
Drinker	4 (12.12)	15 (17.44)	0.478	7 (18.42)	8 (16.67)	1.000
History of atrial fibrillation	0 (0)	4 (4.70)	–	3 (6.30)	1 (2.60)	0.416
Family history of coronary artery disease	0 (0)	0 (0)	–	0 (0)	0 (0)	–
**Medications, n, %**						
Beta-blocker	0 (0)	53 (61.60)	–	23 (60.50)	30 (62.50)	1.000
Ca-channel blocker	0 (0)	36 (41.86)	–	16 (42.11)	20 (41.67)	0.570
ACE inhibitor or ARB	0 (0)	31 (36.05)	–	14 (36.84)	17 (35.42)	1.000
Diuretic	0 (0)	23 (26.74)	–	6 (15.79)	17 (35.42)	0.051
Trimetazidine	0 (0)	22 (25.58)	–	6 (38.00)	16 (48.00)	0.083
Digoxin	0 (0)	1 (1.16)	–	0 (0)	1 (2.08)	1.000
﻿Serum creatinine (mol/L)	–	79.69 ± 16.11	–	80.11 ± 16.85	79.16 ± 15.39	0.808
**Biomarkers**						
Aspartate aminotransferase (U/L)	–	29.34 ± 21.16		27.52 ± 18.08	30.51 ± 23.07	0.469
Creatine kinase (U/L)	–	103.00 (71.00 – 143.75)		124.00 (84.50 – 170.00)	89.00 (68.00 – 136.00)	0.071
Lactate dehydrogenase (U/L)	–	208.03 ± 61.79		212.96 ± 55.27	204.87 ± 66.13	0.378
Creatine kinase-MB (ng/mL)	–	2.30 ± 2.60		1.80 ± 1.72	2.63 ± 3.02	0.038
hs-cTnI (ng/L)	–	12.70 (6.80 – 31.40)		5.60 (3.50–23.30)	21.85 (10.63–39.70)	< 0.001
Nt-proBNP (pg/mL)	–	112.95 (46.45–335.73)		56.85 (18.63–104.43)	220.15 (84.00–420.40)	0.003
> 100.00	–	45 (52.30)		15 (39.50)	30 (62.50)	0.034
≤ 100.00	–	41 (47.70)		23 (69.50)	18 (37.50)	–

Values expressed as n (%) or mean ± SD or median (interquartile range). The *p* values reflect the comparison between 2 groups (controls vs. All HCM). The *p’* values reflect the comparison between 3 subgroups (controls vs. LGE (−) vs. LGE ( +)) or 2 subgroups (LGE (−) vs. LGE ( +)), respectively. LGE, late gadolinium enhancement; LVOTG, peak left ventricular outflow tract gradient; NYHA, New York heart association; ACE, angiotensin-converting enzyme; ARB, angiotensin-receptor blocker; Nt-proBNP, N-terminal pro b-type natriuretic peptide; hs-cTnI, high-sensitivity cardiac troponin I. * < 0.05 vs. controls. ^#^ < 0.05 vs. LGE (−).

### CMR parameters

Table 2 shows the CMR parameters of the study population. The LVEF of all HCM patients was more than 50% (range from 50.83 to 77.73%). Among the 48 LGE-positive patients, the mean LGE% was 10.22 ± 6.24%. Based on the LV myocardial systolic strain analysis, all HCM patients had a significantly lower global peak systolic longitudinal strain (GLS), circumferential strain (GCS) and radial strain (GRS) than the healthy controls (*p* < 0.05 for all). Additionally, the GLS and GCS were significantly lower in the LGE-positive patients than the LGE-negative group and the healthy controls (*p* < 0.05 for all). There were no significant differences in GLS, GCS and GRS between the LGE-negative patients and the healthy controls. The differences in any other LV volume and function parameters are shown in Table 2.

**Table 2:** CMR parameters of the study population.

Variables	Controls (n = 33)	All HCM (n = 86)	*p* value	LGE negative (n = 38)	LGE positive (n = 48)	*p’* value
**Left ventricular function and strains**						
Ejection fraction (%)	57.41 ± 5.47	65.21 ± 6.25	0.327	66.38 ± 5.78*	64.29 ± 6.56*	< 0.001
EDVI (mL/m^2^)	57.15 ± 9.14	65.52 ± 16.48	0.017	59.58 ± 13.48*	70.23 ± 17.23*	< 0.001
ESVI (mL/m^2^)	24.59 ± 5.60	22.99 ± 7.79	0.302	20.15 ± 6.01*	25.23 ± 8.34*^#^	0.004
Stroke volume index (mL/m2)	32.56 ± 4.43	42.53 ± 10.72	< 0.001	39.43 ± 9.18*	44.99 ± 11.29*	< 0.001
﻿Cardiac index (L/min/m^2^)	2.10 ± 0.38	2.72 ± 0.76	< 0.001	2.53 ± 0.69*	2.87 ± 0.78*	< 0.001
﻿Myocardial mass index (g/m^2^)	42.90 ± 5.94	78.97 ± 30.06	< 0.001	64.21 ± 18.71	90.67 ± 40.39*^#^	< 0.001
MWT (mm)	9.16 ± 1.13	18.17 ± 4.34	< 0.001	16.09 ± 3.47*	19.82 ± 4.27*^#^	< 0.001
﻿GLS (%)	− 14.77 ± 2.36	− 11.55 ± 3.70	< 0.001	− 13.43 ± 2.97	− 10.11 ± 3.57*^#^	< 0.001
﻿GCS (%)	− 20.92 ± 2.54	− 20.01 ± 3.58	0.036	− 21.53 ± 2.85	− 18.86 ± 3.67*^#^	0.001
﻿GRS (%)	36.58 ± 8.70	33.25 ± 12.74	0.030	35.54 ± 9.10	31.51 ± 14.80	0.055
**Left ventricular-LGE**						
Enhanced volume (mL)	–	–	–	–	16.71 ± 12.30	-
Enhanced Mass (g)	–	–	–	–	17.55 ± 12.92	-
Enhanced Mass (%)	–	–	–	–	10.22 ± 6.24	-

Values expressed as n (%) or mean ± SD. The *p* and *p’* values reflect comparisons between 2 groups (controls. Vs. total) and 3 subgroups (controls. Vs. LGE (−). Vs. LGE (+)), respectively. CMR, cardiovascular magnetic resonance; LGE, late gadolinium enhancement; EDVI, end-diastolic volume index; ESVI, end-systolic volume index; MWT, maximum wall thickness; GLS, global longitudinal strain; GCS, global circumferential strain; GRS global radial strain. * < 0.05 vs. controls. ^#^ < 0.05 vs. LGE (−).

### Correlations of LGE% with clinical and CMR parameters

The results of the univariate and multivariate linear regression analysis of the LGE% and ﻿the baseline clinical and CMR characteristics in HCM patients are described in Table 3. The LGE% was inversely associated with the use of beta-blockers and the GCS and was positively correlated with serum Nt-proBNP level and LV end-diastolic maximum wall thickness (MWT) (*p* < 0.05 for all) (Fig. 1c–f). The independent determinants of the LGE% were the serum Nt-proBNP level (standardized β = 0.630, *p* < 0.001), MWT (standardized β = 0.483, *p* = 0.001), the use of beta-blockers (standardized β = −0.351, *p* = 0.013) and GCS (standardized β = 0.366, *p* = 0.024) (Table 3).

**Table 3:** Univariate and multivariate regression analysis for LGE (LGE%) and the clinical and CMR indicators in HCM patients.

Variables	LGE%	
	Univariate analysis	
	r value	*p* value
Age (years)	0.018	0.905
Male (%)	0.050	0.736
Body mass index (kg/m^2^)	0.010	0.947
Heart rate (bpm)	− 0.178	0.226
LVOTG at rest (mmHg)﻿	− 0.099	0.550
Obstructive (%)	0.017	0.911
﻿NYHA functional (%)	0.046	0.757
Hypertension (%)	− 0.035	0.813
Diabetes (%)	0.065	0.663
Hyperlipidemia (%)	− 0.182	0.222
History of atrial fibrillation	0.221	0.132
Beta-blocker (%)	− 0.383	0.007
﻿Ca-channel blocker (%)	0.096	0.516
ACE inbibitor or ARB (%)	0.024	0.874
Diuretic (%)	0.239	0.102
Trimetazidine (%)	− 0.051	0.730
Digoxin (%)	0.153	0.300
Serum creatinine (mol/L)	0.257	0.114
Aspartate aminotransferase (U/L)	− 0.279	0.085
Creatine kinase (U/L)	0.011	0.946
Lactate dehydrogenase (U/L)	0.053	0.749
Creatine kinase-MB (ng/mL)	0.190	0.323
hs-cTnI (ng/mL)	0.160	0.323
Nt-proBNP (pg/mL)	0.565	0.001
Ejection fraction (%)	− 0.191	0.194
EDVI (mL/m^2^)	− 0.019	0.899
ESVI (mL/m^2^)	0.075	0.613
Stroke volume index (mL/m^2^)	− 0.118	0.423
Cardiac index (L/min/m^2^)	− 0.174	0.237
Myocardial mass index (g/m2)	0.017	0.907
MWT (mm)	0.486	< 0.001
GLS (%)	0.163	0.279
﻿GCS (%)	0.369	0.012
﻿GRS (%)	− 0.260	0.081

	Multivariate analysis	
	Standardized β	*p* value
Beta-blocker (%)	− 0.351	0.013
Nt-proBNP (pg/mL)	0.630	< 0.001
MWT (mm)	0.483	0.001
GCS (%)	0.366	0.024

LGE, late gadolinium enhancement; LVOTG, ﻿peak left ventricular outflow tract gradient; NYHA, New York heart association; ACE, ﻿angiotensin-converting enzyme; ARB, ﻿angiotensin-receptor blocker; hs-cTnI, high-sensitivity cardiac troponin I; Nt-proBNP, N-terminal pro b-type natriuretic peptide; EDVI, ﻿end-diastolic volume index; ESVI, end-systolic volume index; MWT, maximum wall thickness; GLS, global longitudinal strain; GCS, global circumferential strain; GRS, global radial strain.

### Receiver operating characteristic (ROC) curve analysis

**Figure 2:**
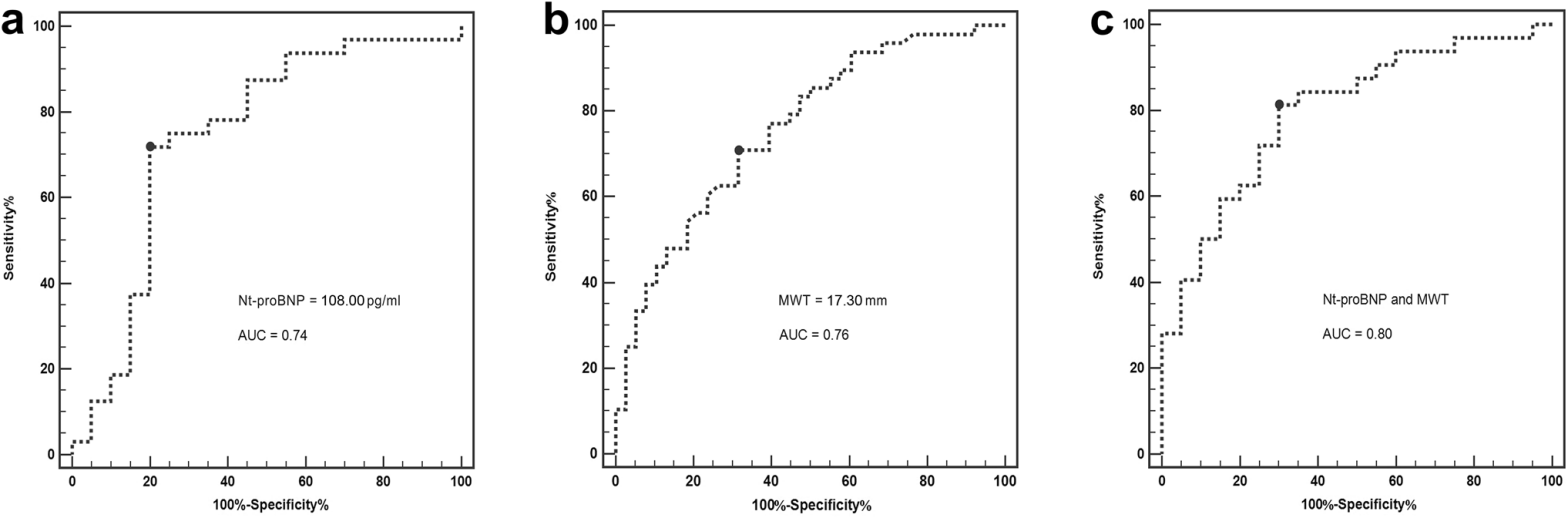
ROC curve analysis of the serum Nt-proBNP levels (**a**), MWT (**b**) and the serum Nt-proBNP level + MWT (**c**) for the identification of the presence of LGE in HCM patients. AUC area under curve, ROC receiver operating characteristic curve analysis, LGE late gadolinium enhancement, HCM hypertrophic cardiomyopathy, MWT Maximum wall thickness, Nt-proBNP N-terminal pro B-type natriuretic peptide.

ROC curve analysis for the detection of the presence of LGE in HCM patients showed an optimal cut-off values of MWT, GCS, and Nt-proBNP were 17.30 mm (*p* < 0.001),  −18.60 (*p* = 0.001) and 108.00 pg/mL (*p* = 0.003), respectively (Table 4). Additionally, the combination of MWT ≥ 17.30 mm with Nt-proBNP ≥ 108.00 pg/mL for the identification of the presence of LGE had a relatively higher area under curve (AUC) (AUC = 0.80), with a sensitivity of 81.25%, a specificity of 70.00%, a positive predictive value (PPV) of 77.38% and a negative predictive value (NPV) of 74.72% (Fig. 2).

**Table 4:** Diagnostic performance of MWT, GCS and Nt-proBNP for identifying the presence of LGE.

Variables	AUC	Sensitivity	Specificity	PPV (%)	NPV (%)	Power
MWT ≥ 17.30 mm	0.76 (0.66–0.86)	70.83 (55.90–83.00)	68.42 (51.30–82.50)	73.91 (58.86–85.73)	64.50 (48.32–79.37)	0.9987
GCS ≥ − 18.60	0.72 (0.61–0.83)	47.83 (32.90–63.10)	88.57 (73.30–96.80)	84.09 (65.09–95.17)	57.34 (43.75–70.17)	0.9736
Nt-proBNP ≥ 108.00 pg/mL	0.74 (0.60–0.86)	71.87 (53.30–86.30)	0.80 (56.30–94.30)	81.94 (67.04–92.08)	69.25 (53.51–82.27)	0.8765
Nt-proBNP + GCS	0.73 (0.59–0.87)	53.33 (34.30–71.70)	89.47 (66.90–98.70)	86.48 (68.90–96.19)	60.28 (46.38–73.07)	0.8951
Nt-proBNP + MWT	0.80 (0.67–0.92)	81.25 (63.60–92.80)	70.0 (45.70–88.10)	77.38 (63.41–87.96)	74.72 (57.39–87.74)	0.9964

MWT, maximum wall thickness; GCS, global circumferential strain; Nt-proBNP, N-terminal pro b-type natriuretic peptide; LGE, late gadolinium enhancement; HCM, hypertrophic cardiomyopathy; AUC, area under curve; PPV, ﻿positive predictive value; NPV, negative predictive value.

### Repeatability analysis

For the repeatability analysis, 20 of the total sample and 15 LGE-positive patients were selected. The intra-class correlation coefficient (ICC) with 95% confidence intervals (CI) was used for assessment of the intra- and inter-observer agreement. The ICC values of the intra-observer agreement for LVEF, GLS, GCS, GRS and the extent of LGE were 0.968, 0.962, 0.947, 0.924 and 0.976, respectively. In addition, the ICC values of the inter-observer agreement for LVEF, GLS, GCS, GRS and the extent of LGE were 0.957, 0.944, 0.928, 0.905 and 0.953, respectively.

## Discussion

The present results ﻿﻿showed that elevated levels of Nt-proBNP were significantly correlated with the extent of LGE and were an independent predictor of the presence of myocardial fibrosis. Similarly, there were two studies reporting that Nt-proBNP levels were higher in LGE-positive HCM patients than in those without it, and demonstrating that Nt-proBNP levels had diagnostic value for detecting LGE^14,15^. Additionally, the proportions of patients with Nt-proBNP values of > 100.00 pg/ml were significantly higher in LGE-positive patients than in LGE-negative group in our study. Circulating Nt-proBNP is primarily produced by cardiomyocytes and is released in response to increased myocardial tension, stretching and neurohormonal activation^22^. Previous research findings implicated that myocardial fibrosis could promote diastolic dysfunction and abnormal microcirculation, leading to ischemia and replacement scarring^23–25^. Additionally, direct Nt-proBNP synthesis by cardiac fibroblasts, as an inhibitory antifibrotic response via the extracellular signal-related kinase pathway has been demonstrated^26^. Thus, there is adequate pathophysiological background to consider investigating the potential of Nt-proBNP as a biomarker reflecting myocardial fibrosis.

Consistent with previous findings^15,27^, we observed that serum levels of hs-cTnI were ﻿significantly higher in LGE-positive HCM patients than in LGE-negative patients. Hs-cTnI is a sensitive and specific biomarker of myocardial ischemia or injury, resulting in myocardial fibrosis^28^. Although hs-cTnI was not a predictor of the presence of LGE in the multivariate analysis in our study, clinicians still should pay attention to this biomarker when it was abnormal. Additionally, we also found that the LGE% was independently associated with MWT, which is in line with previous reports^29,30^. As the presence of LGE could be observed especially in areas of ventricular hypertrophy in HCM patients^31^, the correlation between MWT and myocardial fibrosis is considered reasonable. Moreover, a level of Nt-proBNP ≥ 108.00 pg/mL and MWT ≥ 17.30 mm had excellent diagnostic performance for the detection of LGE on CMR. These results suggest that the measurement of Nt-proBNP and MWT could be a non-invasive method of predicting myocardial fibrosis in HCM patients with preserved EF.

The current study also found that the GLS and GCS were significantly decreased in HCM patients, which was especially true in LGE-positive group. Although the LVEF was normal or increased in the vast majority of HCM patients, the individual cardiac myocyte contractile and stretching forces were damaged and decreased, resulting in intrinsic dysfunction and myocardial remodeling^32,33^. Thus, myocardial systolic strains can detect cardiac dysfunction earlier than LVEF, especially in HCM patients with normal EF. Additionally, in the present study, the GCS is independently associated with LGE%, a finding consistent with a study of HCM patients showing that LGE was strongly correlated with GCS^34^. On the contrary, some previous studies demonstrated that GLS was associated with the extent of LGE^35,36^. A possible explanation of the above differences may be the differences in the post-processing software used, the deformation acquisition techniques, study populations, clinical stages and medications in previous studies. Furthermore, GCS also had diagnostic value for detecting LGE in our study, therefore suggesting its potential clinical utility for reflecting the presence of underlying myocardial fibrosis.

In the present study, LGE positive patients had a trend towards a higher prevalence of diabetes mellitus than in LGE negative patients. The presence of diabetes may have an impact on the features of HCM because of diabetes is correlated with higher degrees of diastolic dysfunction^37^. In addition, LGE negative patients had the highest heart rate among the groups. Although beta-blocker use was similar in both HCM groups, dosage may have been different possibly explaining observed differences in heart rate. Our results also showed that the use of beta-blockers was inversely associated with the amount of LGE. Beta-blockers therapy have proved effective in reducing ﻿myocardial ischemia and LVOT obstruction, and the current guidelines suggested these drugs as first-line treatment in symptomatic patients with HCM^1^. The advantages of beta-blockers are mediated by sympathetic modulation of myocardial contractility, stiffness and heart rate, which can improve myocardial compliance and increase ventricular diastolic filling time^38,39^. Additionally, we observed that patients with LGE have a trend towards a higher use of diuretics and trimetazidine than in those without LGE. This may be related to more severe disease and clinical manifestations of HCM patients with LGE. In HCM patients, diuretics were used to treat hypertension or heart failure^40^, while trimetazidine had anti-ischemic effect^41^. However, the mechanism of pharmacological therapy of HCM is complicated, future interventional studies are warranted.

There are several limitations in the present study. First, the sample size was relatively small. Second, this was a single-centre study, and the HCM subjects were selected with stringent criteria; therefore, some inherent biases were inevitable. Third, as the control group was healthy volunteers, and they had no indicators to do laboratory tests, so the laboratory findings of the controls were unavailable. Lastly, although LGE on CMR is limited to identifying diffuse myocardial fibrosis, this technique is widely and frequently used to assess myocardial fibrosis in different types of cardiovascular diseases. The current study used 6SD thresholding method, previously shown to yield improved interobserver variability, reproducibility and precision with regards to LGE, as well as stronger correlations with histopathology in HCM patients^42,43^. However, future large cohort studies with longer follow-up are needed to further confirm the parameters and predictors of the presence of LGE in HCM patients.

In conclusion, our results show that Nt-proBNP is a useful biomarker for detecting LGE and, combined with MWT, has good diagnostic performance for myocardial fibrosis in HCM patients with preserved EF. Additionally, the decreased LV GCS was independently correlated with the LGE%, indicating its potential prognostic value for detecting myocardial fibrosis. These findings suggesting that the combined non-invasive clinical biomarker and imaging technology may be utilized to predict myocardial fibrosis in HCM patients and, help clinicians to identify patients with poor prognosis at an early stage.

## Materials and methods

### Study design and participants

This study was approved by the ethics committee of Tongji Medical College, Huazhong University of Science and Technology, and all participants in the study signed informed consent forms autonomously and voluntarily prior to participation. All research methods were performed in accordance with the relevant guidelines and regulations.

We prospectively recruited 118 consecutive HCM patients who were referred for CMR examination from January 2018 to June 2019. Thirty-five age- and sex matched healthy subjects who responded to advertisements were recruited to serve as the control group. HCM was diagnosed by CMR with the following criteria: unexplained LV wall thickness ≥ 15 mm (or ≥ 13 mm with a clear family history of HCM) in adult patients without any other systemic disease or cardiac diseases that could be responsible for myocardial hypertrophy^1^. The preserved EF was defined as LVEF ≥ 50% by CMR or echocardiography. Patients with evidence of coronary heart disease (≥ 50% stenosis assessed by computed tomography or invasive coronary angiography), ﻿ischemic cardiomyopathy, valvular heart disease, connective tissue disease, Anderson Fabry disease, LVEF < 50% as measured by CMR or echocardiography, ﻿concomitant neoplasm and infection, or with a history of invasive cardiac procedure, such as alcohol septal ablation, septal myectomy or heart transplantation, were excluded. The inclusion criteria for the controls were no known history of hypertension, diabetes mellitus, hyperlipidemia, or cardiovascular diseases, and they had a normal electrocardiogram findings and normal cardiac function and tissue characterization (without LGE) by CMR. The exclusion criteria for all subjects included renal dysfunction (glomerular filtration rate (eGFR) < 30 mL/min/1.73 m^2^) and any CMR contraindications, such as claustrophobia or inner device implantation. According the above criteria, 19 HCM patients had a LVEF < 50%, 5 patients lacked LGE images because they had renal dysfunction, 4 patients had a history of severe coronary artery disease, 4 patients had a history of cardiac surgery and 2 healthy volunteers had a mild mitral regurgitation. Thus, 86 HCM patients and 33 healthy controls were eventually enrolled in the present study.

### Laboratory measurements

The biochemical indices included the serum Nt-proBNP, hs-cTnI, creatine kinase (CK), CK-MB, aspartate aminotransferase (AST) and lactate dehydrogenase (LDH) levels, which were obtained for clinical evaluation purposes. Peripheral venous blood samples were collected from all HCM patients at the morning of CMR examination. Blood samples were centrifuged at 3000 rpm for 15 min, and plasma was stored at − 80 °C for further analysis. An electrochemiluminescent immunoassay assay (Roche Diagnostics, Mannheim, Germany) was performed for measurement of plasma Nt-proBNP levels. The analytical range was 5 to 35,000 pg/mL and the normal reference range was ≤ 100 pg/mL. The inter-assay and intra-assay coefficients of variation were < 4.7 and < 5.8%, respectively. Serum levels of hs-cTnI were measured using the Abbott Architect high-sensitivity cTnI assay (Abbott Diagnostics, Abbott Park, USA). The lower limit of detection was 1.2 ng/L; the 99th percentile cutoff value was 26 ng/L; and the coefficient of variation was < 10%. Serum CK, CK-MB, AST and LDH levels were determined using an automatic particle chemiluminescence immunoassay (Abbott Aeroset, Minnesota) with the use of commercial kits (Abbott). The normal reference ranges of the assays were the following: 38 to 174 U/L for CK, < 6.6 ng/mL for CK-MB, 8 to 40 U/L for AST and 109 to 245 U/L for LDH.

### CMR examination protocol

All CMR examinations were performed with a 1.5T system (MAGNETOM Aera, Siemens Healthineers, Erlangen, Germany). The LV long-axis (4-, 3-, 2-chamber) and short -axis (covering all basal to apex segments) cine images were acquired using a balanced steady state free precession (bSSFP) sequence. LGE images of the LV long-axis (4-, 3-, 2-chamber) planes and whole LV short-axis slices were performed 10–15 min after the cubital intravenous administration of a bolus of gadolinium-diethylenetriamine pentaacetic acid (DTPA) (0.2 mmol/kg, Magnevist, Bayer Healthcare, Berlin, Germany) using a phase-sensitive inversion recovery (PSIR) sequence. The parameters of LGE scans were as follows: field of view (FOV): 360 mm × 270 mm, matrix: 256 × 192, repetition time/echo time (TR/TE): 12.44 ms/1.19 ms, inversion recovery time: 300 ms; flip angle: 40°, and slice thickness: 8 mm.

### CMR image analysis

**Figure 3:**
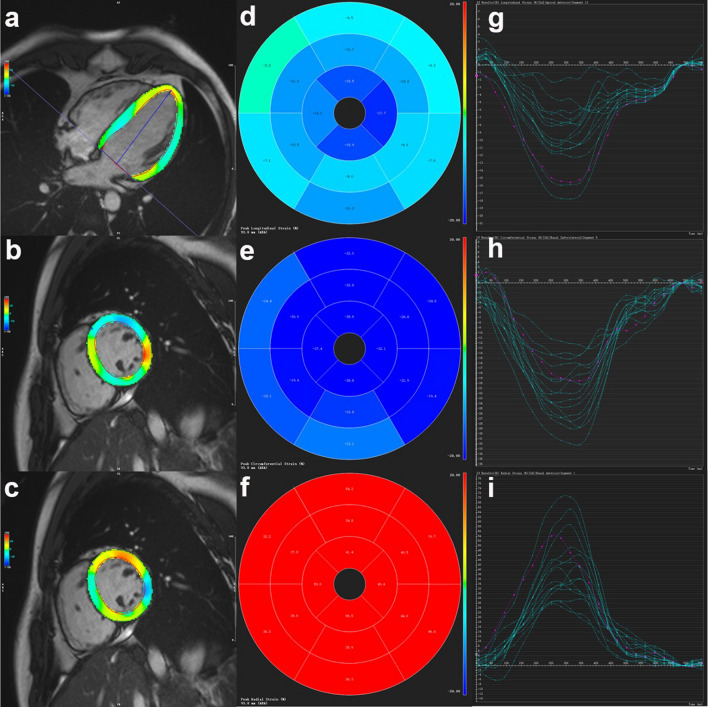
Example of left ventricular myocardium peak systolic strain analysis in a healthy volunteer. The left shows the colored tissue-tracking maps of longitudinal (**a**), circumferential (**b**), and radial (**c**) strain. In the middle is the 16-segment model of the longitudinal (**d**), circumferential (**e**), and radial (**f**) values in a cardiac cycle. On the right are the strain–time curves of the longitudinal (**g**), circumferential (**h**), and radial (**i**) values in a cardiac cycle.

All CMR image analyses were semi-quantitatively performed using the commercial post-processing software (Cvi42, Circle Cardiovascular imaging, Calgary, AB, Canada). The LVEF, end-diastolic volume (EDV), end-systolic volume index (ESV), stroke volume index (SV), cardiac index and end-diastolic myocardial mass were calculated manual delineation of the LV endocardial and epicardial contours in ﻿end-diastole and end-systole using short-axis cines. All cardiac functional parameters were indexed to the body surface area (BSA) in this study. The LV ﻿end-diastolic MWT was defined as the greatest segments of the 16-segment model of the American Heart Association (AHA). ﻿Myocardial systolic strain analysis was quantified ﻿manual delineation of the endocardial and epicardial borders using LV long- and short-axis cines in the end-diastole stage. The LV 3D peak systolic GLS, GCS and GRS were semi-quantitatively calculated using the tissue feature tracking method (Fig. 3).

**Figure 4:**
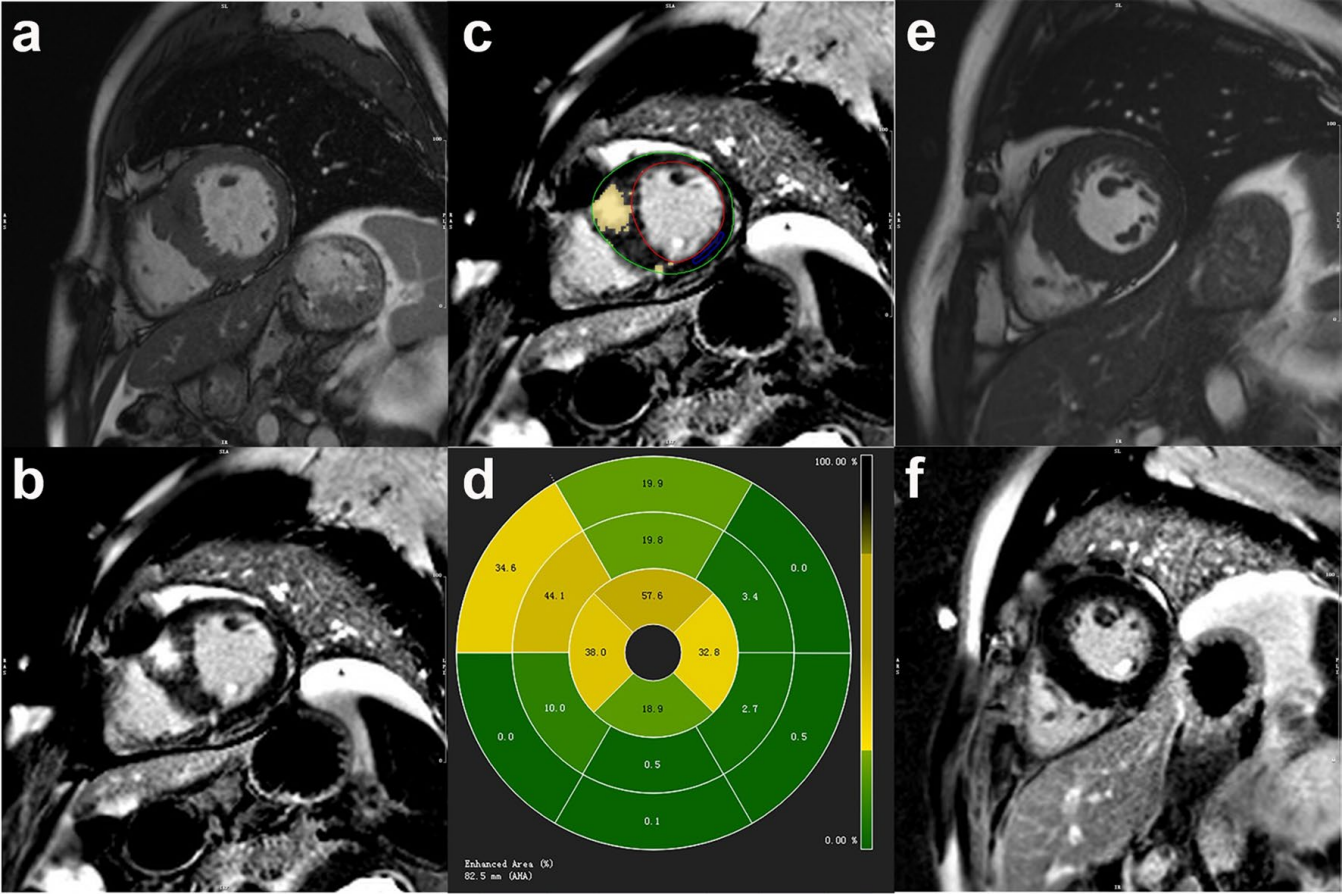
Examples of hypertrophic cardiomyopathy patients with and without LGE. On the left are the short-axis cine (**a**) and LGE images (**b**) in a 46-year-old man. (**c**) Same image showing quantification of LGE using the 6SD thresholding method. (**d**) A representative 16-segment model of the LGE% in this patient. On the right are the short-axis cine (**e**) and LGE images (**f**) in a 46-year-old man without LGE. LGE late gadolinium enhancement.

For the quantification of the extent of LGE, we imported the whole LV short-axis slices of the LGE images into the software and manually delineated the LV endocardial and epicardial contours. The enhanced myocardium was defined as a signal intensity threshold of > 6 standard deviation (SD) above the mean signal intensity of the normal myocardium^43^. The total LV enhanced volume and mass were calculated, and the extent of LGE was expressed as the percentage of the total myocardial mass (%LGE) (Fig. 4). The HCM patients were divided into two subgroups based on the presence or absence of LGE. All papillary muscles and trabeculae were excluded from the LV myocardium during the LV function, deformation and LGE analyses.

### Statistical analysis

The Kolmogorov–Smirnov test was used to check normality. Data are expressed as the mean ± SD or number (percentage) or median (interquartile range), as appropriate. Differences between two groups were assessed using an independent-sample Student’s t test or the Mann–Whitney U test. Comparisons between three groups were analyzed using one-way ANOVA or the Kruskal–Wallis test, and the Bonferroni correction was selected as the post hoc test when appropriate. The chi-square test or Fisher’s exact test was used for the comparison of all categorical variables. Pearson’s or Spearman’s correlation test was applied for the assessment of the LGE% and all candidate variables. Univariate and multivariate linear regression analyses were utilized to assess the associations between the LGE% and all candidate variables. We chose age, diabetes, Beta-blocker, diuretic, hs-cTnI, Nt-proBNP, MWT, GLS and GCS as the nine variables included in the final multivariate analysis using a stepwise algorithm model. The baseline variables included in the final models were based on clinical and scientific constraints, and the results of univariable analyses. ROC curve analysis was applied for the detection of the diagnostic performance of the presence of LGE. A two-sided *p* value < 0.05 was considered statistically significant. Analyses were performed using SAS (SAS, version 9.4, SAS Institute, Cary, NC, USA), and MedCalc 16.2.0 (MedCalc Software, Mariakerke, Belgium).

## Ethics declarations

This study was approved by the ethics committee of Tongji Medical College, Huazhong University of Science and Technology, and all participants in the study signed informed consent forms autonomously and voluntarily prior to participation. All procedures performed in studies involving human participants were in accordance with the ethical standards of the institutional and/or national research committee and with the 1964 Helsinki declaration and its later amendments or comparable ethical standards.

## Data Availability

The datasets used and analyzed during the current study are available from the corresponding author on reasonable request.
